# Traumatic Brain Injury and Neuromodulation Techniques in Rehabilitation: A Scoping Review

**DOI:** 10.3390/biomedicines12020438

**Published:** 2024-02-16

**Authors:** Andrea Calderone, Davide Cardile, Antonio Gangemi, Rosaria De Luca, Angelo Quartarone, Francesco Corallo, Rocco Salvatore Calabrò

**Affiliations:** IRCCS Centro Neurolesi Bonino-Pulejo, S.S. 113 Via Palermo, C. da Casazza; 98124 Messina, Italy; andrea.calderone95@gmail.com (A.C.); antonio.gangemi@irccsme.it (A.G.); rosaria.deluca@irccsme.it (R.D.L.); angeloquartarone@irccsme.it (A.Q.); francesco.corallo@irccsme.it (F.C.); roccos.calabro@irccsme.it (R.S.C.)

**Keywords:** neuromodulation, traumatic brain injuries, rehabilitation

## Abstract

Background and Objectives: Traumatic Brain Injury (TBI) is a condition in which an external force, usually a violent blow to the head, causes functional impairment in the brain. Neuromodulation techniques are thought to restore altered function in the brain, resulting in improved function and reduced symptoms. Brain stimulation can alter the firing of neurons, boost synaptic strength, alter neurotransmitters and excitotoxicity, and modify the connections in their neural networks. All these are potential effects on brain activity. Accordingly, this is a promising therapy for TBI. These techniques are flexible because they can target different brain areas and vary in frequency and amplitude. This review aims to investigate the recent literature about neuromodulation techniques used in the rehabilitation of TBI patients. Materials and Methods: The identification of studies was made possible by conducting online searches on PubMed, Web of Science, Cochrane, Embase, and Scopus databases. Studies published between 2013 and 2023 were selected. This review has been registered on OSF (JEP3S). Results: We have found that neuromodulation techniques can improve the rehabilitation process for TBI patients in several ways. Transcranial Magnetic Stimulation (TMS) can improve cognitive functions such as recall ability, neural substrates, and overall improved performance on neuropsychological tests. Repetitive TMS has the potential to increase neural connections in many TBI patients but not in all patients, such as those with chronic diffuse axonal damage.Conclusions: This review has demonstrated that neuromodulation techniques are promising instruments in the rehabilitation field, including those affected by TBI. The efficacy of neuromodulation can have a significant impact on their lives and improve functional outcomes for TBI patients.

## 1. Introduction

Traumatic brain injury (TBI) is a condition in which an external force, usually a violent blow to the head, causes functional impairment and structural damage to the brain. It can be caused by a sudden acceleration–deceleration, a blow, a bump, or a collision to the head. This condition can also arise if something breaks or enters the skull. This allows a distinction between open and closed injuries, with the former generally being associated with worse outcomes [1,2]. Sensorimotor deficits, cognitive deficit deterioration, behavioral disorders, depression, and headaches are among the severe long-term effects of trauma on young people worldwide, making it one of the leading causes of death and disability [3,4]. TBI is most commonly experienced in individuals aged 0 to 4, with adolescents aged 15 to 24 being the most susceptible, and it occurs most frequently due to falls and car accidents [5]. The progression of TBI is a multifaceted process that includes primary and secondary injuries, leading to temporary or permanent neurological damage. The secondary injury occurs within minutes to days after the primary impact and consists of an inflammatory cascade that causes further brain damage. In the evaluation of patients with TBI, a detailed neurologic examination should be performed by trauma or emergency department personnel, also using the Glasgow Coma Scale. This is of great importance in this context, as this scale often dictates management according to current guidelines [6]. Rehabilitation, cognitive correction, exercise, and cognitive-enhancing drugs, as well as various brain stimulation techniques, are currently used for treating TBI [7,8].

A growing number of TBI nonpharmacological treatment options include different neuromodulation interventions. A broad spectrum of intervention techniques are included in neuromodulation, which aims to alter nervous system pathological activities to achieve therapeutic effects.

Neuromodulation is a technique used to stimulate the scalp and skull to promote neuronal plasticity and the recovery of neurological processes. In recent years, neuromodulation has evolved, and it is now universally considered an accepted treatment for a variety of neurological and psychiatric disorders [9,10], In particular, two noninvasive methods of neuromodulation provide electrical stimulation to different areas of the brain: transcranial magnetic stimulation (TMS) and transcranial direct current stimulation (tDCS). In TMS, a magnetic field generated by a coil produces a short-lasting electrical current pulse into the brain, especially in the cerebral cortex where neurons are stimulated [11,12]. TMS is classified into three main types: a single pulse, which involves applying pulses at varying intervals every few seconds, a paired pulsed, and repetitive TMS (i.e., rTMS, where magnetic pulses are delivered in rapid succession). TMS has a duration that can either be excitatory (>5 Hz) or inhibitory (>1 Hz), depending on the frequency used [13,14,15].

tDCS alters the resting membrane potential of neurons and affects their spontaneous firing rate [11]. By connecting two or more electrodes to the scalp, a weak direct current is delivered to the skull to reach the cerebral cortex.

Depolarization or hyperpolarizing occurs in neurons’ membranes of the target area as a result of the current entering the brain at the anode and exiting at its cathodes [16,17]. Other techniques are deep brain stimulation (DBS), spinal cord stimulation (SCS), transcutaneous electrical nerve stimulation (TENS), and low-level laser therapy (LLLT) [18,19].

Neuromodulation techniques are thought to restore altered function in the brain, resulting in improved function and reduced symptoms. Brain stimulation can alter the firing of neurons, boost synaptic strength, alter neurotransmitters and excitotoxicity, and modify the connections in their neural networks. All these are potential effects on brain activity. Accordingly, this is a promising therapy for TBI [20]. These techniques are flexible because they can target different areas of the cortex, vary in frequency and amplitude, and are critically noninvasive. Neurological responses to different receptors, neurotransmitter systems, or ion channels are thought by noninvasive brain stimulation to activate multiple neural mechanisms dependent on the task. Obviously, the target area of these techniques changes depending on the function or symptom to be treated [21,22]. Moreover, neuromodulation can modulate neural activity in a way that produces changes in theta, delta, and gamma oscillations [23]. In the neurorehabilitation field, one of the key points for effective neuromodulation techniques, such as TMS, is the correct positioning of the coil in the target cortical area. Neuronavigation helps with the correct application by using brain coordinates obtained from the patient’s magnetic resonance imaging (MRI) slices. A three-dimensional reference system is used to transfer the coil and head coordinates, which are determined by the optical camera of the patient tracker, infrared position sensor, and coil trackers. Through this method, the brain’s specific stimulation area can be monitored while using a computer screen [24].

Regarding TBI, there are clear opportunities and challenges to the practical application of neurostimulation techniques, including TMS, tDCS, or DBS.

For example, there is a landmark case study in which central thalamic DBS induced the recovery of a patient in a persistent minimally conscious state [25]. However, a subsequent study of 14 patients found that patients with improved outcomes had more intact postinjury neuroanatomical structures, whereas improvement was not observed in those with larger lesions [26].

TBI patients often exhibit recurrent depressive episodes and invaliding depressive and anxiety symptoms as measured by different clinical instruments (depression questionnaire/scales), and DBS has shown promising results in these behavioral alterations focused on depression signs in both human and animal models and may therefore be a useful treatment option [27]. In addition, other potential cognitive and behavioral sequelae of TBI, such as memory and arousal disorders, may also be indications for DBS [28].

TMS and tDCS have specific effects on the rehabilitation of TBI patients. TMS has been used to treat major depression [27], schizophrenia, Parkinson’s disease, aphasia, unilateral neglect, cognitive impairment, and other related conditions. The FDA has also approved its use for these conditions [29]. Patients who undergo TBI have reported significant improvements in their cognitive function and depression, as evidenced by the successful delivery of repetitive high-frequency (10 Hz) TMS to the left dorsolateral prefrontal cortex [30].

Moreover, TMS and tDCS can decrease symptoms related to TBI (tinnitus, neglect, memory impairment, and attention deficit disorder) and lead to significant improvements in the upper extremities on the Fugl–Meyer scale [31,32].

Another technique is vagus nerve stimulation (VNS). Afferent and efferent fibers are transported between the medulla oblongata and organs in the chest and abdomen through the vagus nerve, which is the 10th cranial nerve. This process involves moving these fibers from one body to another. The vagus nerve, which is the parasympathetic branch of the autonomic nervous system, is responsible for transmitting sensory information between organs [33,34,35]. We have descripted these main neuromodulation techniques in the Table 1. 

This scoping review aims to investigate the recent literature of the last years about neuromodulation techniques used in the rehabilitation of TBI patients.

## 2. Materials and Methods

### 2.1. Search Strategy

**Figure 1:**
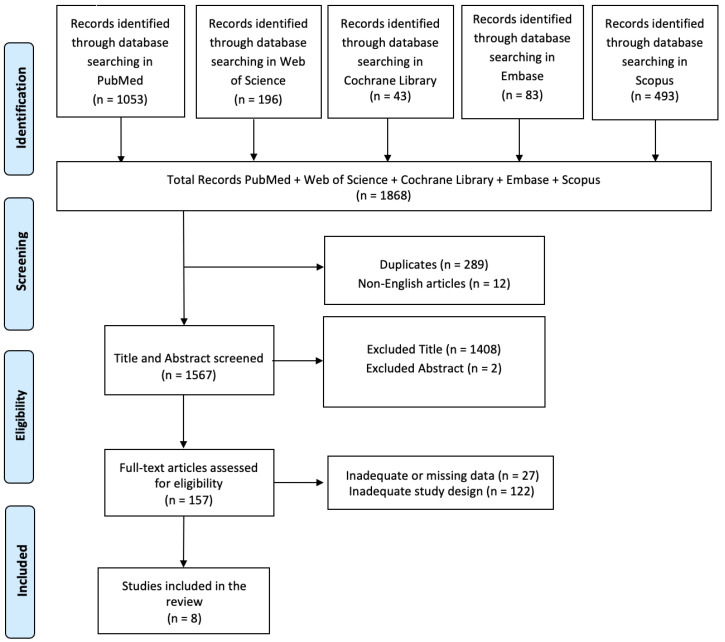
PRISMA 2020 flow diagram of evaluated studies.

A literature search was conducted via PubMed, Web of Science, Cochrane Library, Embase, and Scopus, and it was carried out for articles using the following search string: (Neuromodulation) AND (traumatic brain injuries); with 2013–2023 search time range. We adopted the PRISMA (Preferred Reporting Items for Systematic Reviews and Meta-Analyses) flow diagram to describe the sequence of steps (identification, screening, eligibility, and inclusion) for the collection and determination of qualified studies, as shown in Figure 1. Titles and abstracts were independently scanned and retrieved from database searches. The suitability of the article was then assessed according to the defined inclusion criteria. Ultimately, we received all titles and abstracts that met the criteria for inclusion in the full text. To avoid bias, several expert teams worked together, selected the articles, analyzed the data independently, and discussed any discrepancies with each other. Disagreements between reviewers were resolved by consensus. This review has been registered on OSF (JEP3S).

### 2.2. PICO Evaluation

We defined our combination of search terms using a PICO (population, intervention, comparison, outcome) model. The population was limited to patients with moderate to severe TBI; the intervention included all studies, rehabilitation approaches, electrical and magnetic brain/nerve/spinal cord stimulation in the field of rehabilitation, and those used to measure and assess TBI patients; the comparison was evaluated considering the different instruments and neuromodulation techniques that produced some data or effects in patients with TBI both before and during rehabilitation process; and the result included any data or improvements of these patients during the rehabilitation process.

### 2.3. Inclusion Criteria

A study was included if it described or investigated neuromodulation techniques used in the rehabilitation of TBI patients. This review included only articles written in English. Clinical studies and trials describing or investigating the functional assessment of these patients were also included. Case reports were not taken into account. We only included studies conducted in human populations and published in English that met the following criteria: (i) original or protocol studies and (ii) articles that tested the effects of neuromodulation used in TBI patients as a rehabilitation strategy.

### 2.4. Exclusion Criteria

A study was excluded if there was a lack of data or information about the description of a neuromodulation intervention used in the rehabilitation of TBI patients. Systematic, integrated, or narrative reviews were also excluded, but reference lists were reviewed and included as necessary. All articles written in languages other than English were excluded.

## 3. Results

In total, 1868 articles were found: A total of 289 articles were removed due to duplication after screening; 12 articles were excluded because they were not published in English; and 1410 articles were excluded based on title and abstract screening. Finally, 149 articles were removed based on screening for inadequate study designs and untraceable articles (Figure 1). The review includes eight research articles considered eligible. A summary of these studies is shown in Table 2.

The articles described in this review investigated neuromodulation techniques used in the rehabilitation of TBI patients. The neuromodulation techniques used in the rehabilitation of TBI patients were analyzed in eight articles [53,54,55,56,57,58,59,60].

### Neuromodulation Techniques and Rehabilitation in TBI Patients

The contemporary field of neuromodulation could have significant implications in the field of rehabilitation of TBI patients. In one study, personalized machine learning classifiers were trained to predict moment-to-moment changes in memory function in each TBI patient by analyzing neural data across electrodes as the patient learned and recalled a list of words. High-frequency stimulation of the temporal cortex was delivered by these classifiers during the predicted memory loss. The strategy demonstrated a 19% improvement in retention of stimulated lists compared to unstimulated ones (*p* = 0.012). Kahana et al. stated that closed-loop brain stimulation could be a potential solution for treating memory impairment caused by TBI. The effectiveness of computer-based cognitive training in augmenting episodic memory with active tDCS was evaluated to compare it to sham tDCS. Alpha frequency was shown to increase near active electrodes, demonstrating better performance correlation in neuropsychological tests [52]. In addition, Sacco et al. [60] displayed that cognitive effort is reduced in patients with TBI through combined tDCS and computer-based training, which appears to promote neuronal reorganization. In another randomized clinical trial in TBI patients, 20-minute tDCS sessions were administered concurrently with computer-assisted cognitive training (20 min) for 10 days (2 weeks, excluding Saturdays and Sundays). Patients were assessed at baseline (T0), at the end of the last stimulation session (T1), and three months after the last tDCS session (T2). The results showed decreased delta activity and increased alpha frequencies around the active electrode with better performance correlates in neuropsychological tests [55]. There is some caution, but evidence suggests that tDCS is safe and cognitively effective at all levels of TBI acuteness and severity [61,62]. Both TMS and tDCS have been shown to slightly affect working memory through transdiagnostic processes, according to recent evidence. tDCS also improved attention and vigilance across all diagnoses. In contrast, Lesniak et al. did not provide sufficient evidence for the efficacy of repetitive A-tDCS to improve memory and attention rehabilitation in patients after severe TBI [8]. Furthermore, functional MRI and spectrally dynamic model were used to examine changes in brain active connectivity before and after administration of high-frequency (10 Hz) neuroimaging (rTMS) targeting the left dorsolateral prefrontal cortex. Following neuromodulation, excitatory connections showed a decrease in strength, and inhibitory links showed an increase. Changes in connectivity between the dorsal anterior cingulate cortex, the left anterior insula, and the medial prefrontal cortex after rTMS administration may be a potential neural mechanism underlying improvements in emotional health [56]. In a randomized clinical trial, high-frequency repetitive TMS was used in cognition rehabilitation in patients with severe TBI and chronic diffuse axonal injury. Between-group comparison of Trail Making Test Part B performance at baseline and after the 10th repetitive TMS session showed no difference between groups (*p* = 0.680 and *p* = 0.341, respectively). No significant differences were found in other neuropsychological tests, nor were adverse events observed between treatment groups, suggesting that high-frequency repetitive TMS does not improve cognitive function in people with chronic diffuse axonal injury [57]. In another study, TMS was combined with electroencephalography in patients with TBI. The inhibitory effect of continuous theta burst stimulation was significantly increased, and N45 modulation was significantly correlated with time since injury in patients, indicating plasticity in the inhibitory network containing γ-aminobutyric acid [58]. A research study in patients with mild to moderate TBI demonstrated that translingual nerve stimulation produced behavioral changes in sensory organization tests and dynamic gait indices. Analyses revealed increased resting functional connectivity between the left inferior parietal lobule of the left postcentral gyrus and the left Brodmann area 40 and increased resting functional connectivity between the right culmen and right declive, demonstrating changes due to translingual nerve stimulation treatment. However, no correlation was found between sensory/somatomotor (visual or cerebellar) networks and sensory organization test/dynamic gait index behavioral performance [59]. Another study compared the efficacy of high-frequency and low-frequency noninvasive translingual neurostimulation with targeted physiotherapy for the treatment of chronic balance and gait disorders due to mild to moderate TBI. It was found that both groups (high-frequency pulse + physiotherapy group and low-frequency pulse + physiotherapy group) maintained improvements in balance scores and outcomes for 12 weeks after discontinuation of translingual neurostimulation treatment [60]. In a last randomized clinical trial, transcranial LLLT was performed within 72 h of trauma using a custom-made helmet. MRI was performed during the acute (within 72 h), early subacute (2–3 weeks), and late subacute (approximately 3 months) recovery phases. Of the 68 randomized patients (33 in the LLLT group and 35 in the sham treatment group), 28 completed at least one LLLT session. Radial diffusivity, mean diffusivity, and fractional anisotropy showed a significant time–treatment interaction at 3 months, indicating that light therapy involves neural substrates involved in the pathophysiological factors of moderate TBI and suggesting diffusion imaging as a biomarker of treatment response [54].

## 4. Discussion

Our review aimed to analyze the recent literature of the last ten years about the neuromodulation techniques used in the rehabilitation of TBI patients.

The studies included in this review have demonstrated that neuromodulation techniques can improve the rehabilitation process for TBI patients in several ways.

tDCS is considered a tool of minimal risk by the Food and Drug Administration to be used use in people with neurological impairments and especially in those with psychiatric symptoms, such as depression [63]. Indeed, noninvasive brain neurostimulation has proven promising to enhance attention deficits in patients with TBI [62], as well as other cognitive domains. Begemann M.J. et al. 2020 discovered a minor yet noteworthy impact on working memory in brain injury patients with TMS and tDCS. Attention/vigilance enhancement was found to be more effective with tDCS than with other forms of treatment [62]. In line with these considerations about the role of neuromodulation techniques on TBI’s cognitive functions, TMS can improve cognitive functions such as recall ability, neural substrates, and overall improved performance on neuropsychological tests [54,55,56]. Repetitive TMS has the potential to increase neural connections in many TBI patients but not in all patients, such as those with chronic diffuse axonal damage. However, this method can be used in combination with other methods, such as electroencephalography, to stimulate plastic processes in specific networks, such as inhibitory networks [56,57,58]. Furthermore, the translingual nerve stimulation method can also be used to stimulate the left posterior parietal gyrus, left inferior parietal lobule, and left Brodmann’s area, along with balance capacity and may increase their functional connectivity and capacity (even several weeks after intervention) [59,60].

### Perspective and Neuromodulation

The scientific literature supports that in healthy controls, neurologically and psychiatrically impaired individuals with repeated high- and low-frequency TMS can induce changes in cortical excitability beyond the duration of stimulation. Repetitive TMS is a crucial indication of its potential to promote neuroplasticity and/or neural adaptation as incorporated therapeutic interventions [64]. Since high-frequency repetitive TMS has shown favorable effects in other populations with reduced cortical motor excitability, this similarity suggests that applying repetitive TMS to cortical motor areas may be beneficial for TBI patients. Rehabilitation in this population may prioritize restoring consciousness rather than voluntary motor function. Thus, it could be more appropriate to concentrate on other areas of the brain, like the prefrontal cortex, to stimulate consciousness. Nonspecific activation impulses and specific sensory input are both important components of cortical activity. These activation impulses are generated from the reticular formation of the brainstem, medulla, cortex, and midbrain. Although the reticular activating systems for ascending and descending are well-integrated, the latter is typically located in the central part of the cortex and midbrain, while the former is more commonly found in its central region within the entire cortex [65]. For example, another area that can be stimulated to help TBI patients recover better is the trigeminal nerve. It is the biggest cranial nerve and has considerable connections in the central nervous system. The trigeminal nerve projects directly or indirectly through the ascending reticular activating system to subcortical structures, the spinal locus, and the cortex [66]. Trigeminal nerve stimulation is a novel noninvasive neuromodulatory treatment for a variety of functional brain disorders, including drug-resistant epilepsy [67,68], major depressive disorder [69,70], and attention deficit hyperactivity disorder [71]. Previous studies have shown that trigeminal nerve stimulation successfully awakens unconscious patients [72] and that activation of the trigeminal spinal nucleus and lateral hypothalamic neurons can facilitate recovery from TBI-induced comas [73].

Evidence suggests that neuromodulation has the potential to modify theta activity in humans. As an illustration, cognitive training is complemented by various neuromodulatory techniques that can be beneficial for healthy and clinical populations [74,75,76,77]. The high prevalence of people suffering from persistent TBI-related cognitive impairment, the lack of research examining tDCS on cognition after TBI, and the lack of research investigating tDCS to inform TBI science and applications provide the need for data elements that reflect current evidence regarding the use of tDCS for cognition after TBI. Furthermore, combining tDCS with existing treatments improves functional outcomes [78]. The combined approach can elicit task transfer [79,80], a sought outcome in rehabilitation. A combination of neuroimaging and neuromodulation is useful to identify the mechanisms underlying recovery. Studies combining electroencephalography and tDCS have found that improvements in working memory are due to improvements in theta attributes such as phase synchronization, phase–amplitude coupling, and theta–gamma cross-frequency coupling [81,82]. Theta synchronization can be improved through transcranial alternating current [83], but the individual’s frequency needs to be adjusted [84]. Determining how neuromodulation can realign theta attributes and other neural patterns holds promise in TBI because previous research displays that, after tDCS, TBI patients showed superior cognitive outcomes [85]. Photobiomodulation using LLLT has been tested as a new technique to optimize recovery of patients with traumatic brain injury (TBI) and was shown by Poiani et al. (2018) in their randomized double-blinded trial as improving the memory, attention, and mood in healthy and neurologic patients [51]. 

Furthermore, it is well known that direct stimulation of the cortex produces neuroplasticity similar to those induced by rehabilitation training. Both suprathreshold and subthreshold electrical stimulation can strengthen synaptic connections and trigger neuronal reorganization, leading to functional and cognitive improvements. Despite the promising results and benefits, the disadvantages of these innovative technologies need to be carefully analyzed to plan and organize the rehabilitation process functionally and effectively.

## 5. Neuromodulations’ Disadvantages and Limitations

Despite recent advances in TMS and the plethora of studies conducted, several physiology, engineering, and clinical challenges remain regarding the use of neuromodulation as a treatment for clinical conditions [86]. These limitations affect both external and internal stimulation techniques. Though these principles may be simple, the actual shape of the induced current TMS is often not very clear because there are variations in the intracranial anatomy. The preferential flow of current may occur toward areas of cerebrospinal fluid with high conductivity, and the exact location of the current may vary greatly [87,88]. Many patients with TBI have undergone craniotomy, craniectomy, or other neurosurgical treatments, and cranial defects or cranial plaques are common in patients who benefit from neurostimulation therapy [89]. Cranial defects are also common in patients with TBI. Various plate sizes and defects in tDCS can alter the direction of current flow, and finite element modeling has revealed variations in the current distribution across brain regions [90]. However, noninvasive techniques have a significant disadvantage in terms of limited access to the exact structures within the brain. Problems such as the passage of currents through the scalp and cerebrospinal fluid make it difficult to predict or control the targeting of deep structures. Similarly, the electric field generated by TMS is significantly reduced for deeper targets [91]. Studies conducted on coil design have demonstrated that larger coils are necessary to reach deeper structures. The response is less targeted as the coil size increases, resulting in a larger tissue area. In addition, tDCS and TMS are both short-term stimulation methods, and the effects of noninvasive stimulation fade away after months or years. Due to these limitations, stimulation methods that can directly access both superficial and deep structures and stimulate nerves continuously over an extended period should be preferred [92].

## 6. Study Strengths and Limitations

This scoping review has several strengths. It is based on evidence from studies that use neuromodulation techniques specifically for TBI patients. It includes a description of some neuromodulation instruments used in rehabilitation. We have also identified data gaps in many areas, hopefully providing information for future research. The main limitation of the present study is the few papers that meet the inclusion criteria, as we included only eight articles that explored the neuromodulation techniques used in the rehabilitation of TBI. This, in addition to the heterogenous methodology and samples, prevents us from drawing robust evidence on this important topic. The articles were restricted by date, so it is possible that important evidence was omitted. Furthermore, the sample size varies: some are large, some are small, and the parameters measured are different. Although the neuromodulation techniques studied have not yet been shown to be effective in reducing symptoms after TBI, the initial results are promising.

## 7. Future Directions

In our opinion, the implementation of tDCS protocols in inpatient neurorehabilitation units has its limitations, and recommendations of expert researchers are needed to facilitate translational use in clinical practice. We believe that the actual guidelines that identify methods to support the applications of tDCS studies in patients with TBI are genuinely needed. Additional data to map and monitor the current evidence for using neuromodulation techniques for cognitive and motor recovery after TBI is also required. Guideline recommendations for tDCS-based studies on cognitive outcomes after TBI will provide evidence and findings to enhance complex rehabilitation outcomes, including psychometric, neurophysiological, and functional scores. It not only facilitates translation but also facilitates the use of various combined treatment approaches such as robotics, assisted virtual approach, and training using a computer [93,94,95].

## 8. Conclusions

In conclusion, this review displays that neuromodulation techniques are promising instruments in the rehabilitation field, including those affected by TBI. The efficacy of neuromodulation can have a significant impact on their lives as it is better understood by researchers and clinicians and improves functional outcomes for TBI patients. The importance of recognizing abnormalities in brain networks associated with functional and structural abnormalities in TBI patients is increasingly recognized, given the growing potential of spatially accurate neuromodulation techniques to modulate functional brain networks. Future TBI research should investigate biomarkers of dysfunction in a patient-specific manner using structural and functional neuroimaging studies. Despite the paucity of available evidence, the current understanding of the pathophysiology after TBI and the mechanisms of action of different neuromodulatory modalities warrants the exploration of novel interventions that may eliminate the functional consequences of TBI. Prospective safety studies and well-designed studies in TBI are necessary to confirm the efficacy of noninvasive brain stimulation in promoting recovery and reducing disability while specifying specific neuromodulatory parameters and procedures.

## Figures and Tables

**Table 1:** The description of the main neuromodulation techniques in neurorehabilitation.

Neuromodulation Techniques	Description and Characteristics	Picture
Transcranial Magnetic Stimulation (TMS)	A technique known as transcutaneous magnetic stimulation (TMS) is a noninvasive way to stimulate the brain, producing alternating magnetic fields that change rapidly over time. The extensive capabilities of TMS make it a perfect neurophysiological tool for studying the function of brain regions and their associated networks, as well as studying brain–behavior relationships to identify possible neurobiological substrates of diseases [10]. For single-pulse experiments, monophasic magnetic pulses are commonly used, whereas rTMS experiments usually require biphasic stimulation waveforms due to their lower energy requirements [36]. Low-frequency rTMS studies typically employ a 1 Hz stimulation frequency, with differences in both the intensity and number of pulses during each study, which can suppress the effect. Conversely, high-frequency rTMS (5–250 Hz) is believed to enhance cortical excitability [37].	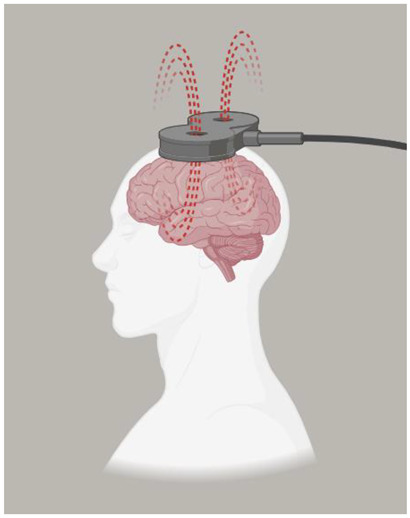 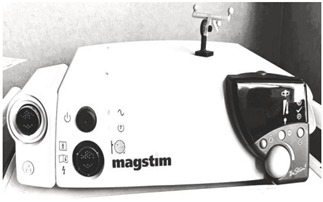 MagstimRapid
Transcranial Direct Current Stimulation (tDCS)	A brain stimulation technique called tDCS delivers low electrical current (2–1 mA) to the cerebral cortex as a means of stimulating cognition and regulating symptoms of neurological disorders and psychiatric. Although common, the side effects include mild itching, burning, and headache, but no lasting effects. A range of approaches can be utilized to pinpoint the location of electrodes. Typically, the 10:20 EEG system is utilized. The measurements can then be used in conjunction with a 10:20 EEG system to localize the region of interest. Alternatively, neuronavigation software, which is more accurate than 10:20 EEG systems, can be used [38]. The scalp can be equipped with electrodes through rubber bands, elastic mesh tubing, or neoprene caps. Keeping the electrodes in place during stimulation is crucial. One study found that as little as 5% movement can change the accuracy and intensity of electrical current to a desired cortical area [39]. The target area (prefrontal cortex, motor cortex, etc.) is stimulated using target electrodes, the location of which depends on the hypothesis and task. Alternatively, hemispheric montages (also known as “dual” stimulation) can be used. In this case, the positioning of both target electrodes is fundamental for downregulation in one region (cathode current) and upregulation in a parallel region (anodic current), opposite hemisphere [40].	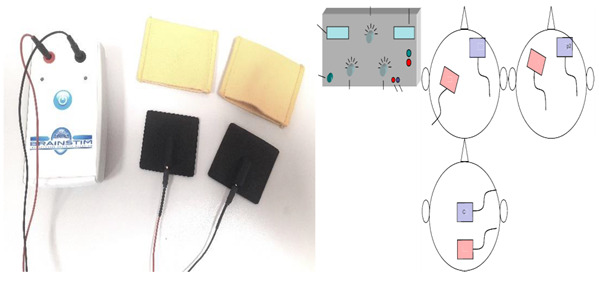 Transcranial electrical stimulator (tDCS)
Vague Nerve Stimulation (VNS)	The VNS is a device that can be implanted, which includes an electrode surrounding the left vagus nerve and an attached unit with batteries and corresponding pulse generators placed under the collarbone. The treatment of drug-resistant depression and epilepsy is often achieved through it, resulting in significant antidepressant and antiepileptic effects. It typically denotes the parametric elements that impact on the administration and delivery of electrical stimulation. It includes: (i) Pulse width is the length of time of a square current pulse. This time parameter is specified in microseconds (μs); (ii) current strength is a measure of the amplitude or strength of an electrical impulse. The unit is milliampere (mA); (iii) frequency is a measure of the total periodic cycles (from the beginning of one pulse to the beginning of the next) in one second. In contrast to the pulse width, the time during which no current is applied is taken into account. This is in Hertz (Hz); (iv) on–off time is the amount of time that pacing and nonstimulation periods are delivered during a specified period. The “on” period is the time during which stimulation with an intensity greater than 0 mA is delivered; (v) during VNS treatment, the duration of time is considered the cumulative timing [41].	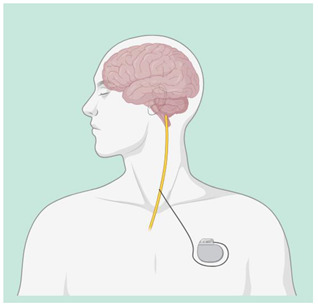 Vagus Nerve Stimulation (VNS)
Deep Brain Stimulation (DBS)	DBS is used through electrodes implanted stereotactically at specific targets in the brain. The electrodes are connected to an implantable pulse generator, which is a pacemaker-like device that is implanted under the skin in the chest wall and typically located beneath the collarbone. A computer, which communicates with the implanted pulse generator via a transcutaneous connection, is used by the clinician to establish stimulation parameters after DBS implantation. Stimulation parameters include electrode contacts that give stimulus amplitude, frequency, and pulse width. In the last years, DBS of various targets has been used to promote recovery in patients with disorders of consciousness with varying results, though evidence supporting the use of DBS in MCS patients following TBI is lacking [42,43].	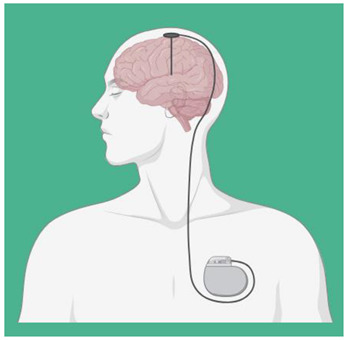
Spinal Cord Stimulation (SCS)	SCS is a form of electrotherapy in which electrodes are implanted into the epidural space of the spinal cord to stimulate the posterior column and modulate nerve function. It is common for the outpatient procedure to last around 1–2 h before a transplant. The surgeon inflates the generator by making an incision, usually on the lower abdomen or buttocks, and then inserts permanent electrodes through a second inlet along one side of the spine after giving local anesthesia. The majority of times, the wound is closed for 2 to 4 weeks after the operation. Advanced leads, advanced remote pulse generators, and traditional SCS are used to treat chronic pain using a variety of stimulation parameters/programs, including high-frequency stimulation, high-frequency burst stimulation, and dorsal root ganglion stimulation [44,45].	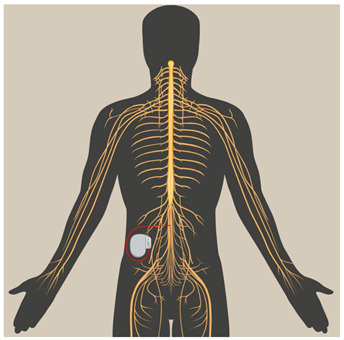
Transcutaneous electrical nerve stimulation (TENS)	The noninvasive TENS method involves the placement of adhesive electrodes on the skin, which deliver pulsed electrical stimulation with a variable frequency, intensity, and duration. The use of it for pain management is widespread in both acute and chronic pain conditions. General battery-powered TENS machines can adjust pulse width, frequency, and intensity. In general, TENS uses high frequencies (>50 Hz) and intensities below motor contractions (sensory intensity) or low frequencies [46,47].	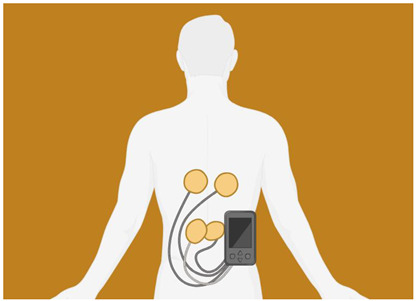
Low Level Laser Therapy (LLLT)	LLLT is a novel noninvasive neurostimulation method that can safely penetrate the brain at specific wavelengths. It is thought to promote cell survival when energy substrates are depleted by interacting with cytochrome c oxidase and promoting oxidative phosphorylation [48,49]. Both animal models and human stroke and TBI patients have reported significant positive effects from LLLT. Kuman et al. showed that LLLT could improve cognitive function in controlled cortical impact (CCI) mice [50]. Poiani et al. [51] used an optical device consisting of an LED emitting 632 nm radiation at full power of 830 mW in patients with TBI. A skull area of 400 cm^2^ was irradiated for 30 min, corresponding to a total dose of 3.74 J/cm^2^ per session.	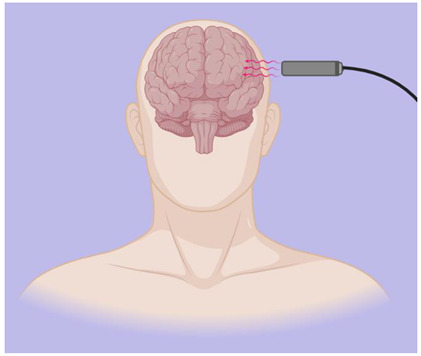

Legend: Transcranial Magnetic Stimulation (TMS), Transcranial Direct Current Stimulation (tDCS), Vague Nerve Stimulation (VNS), Deep Brain Stimulation (DBS), Spinal Cord Stimulation (SCS), Transcutaneous Electrical Nerve Stimulation (TENS), Low-Level Laser Therapy (LLLT).

**Table 2:** Summary of studies included in the research.

Author	Aim	Treatment Period	Sample Size	Outcomes Measures	Main Findings
Kahana et al. 2023 [52]	To assess whether closed-loop tDCS of the temporal lobe cortex can reliably improve memory in a TBI cohort.	1 year.	8 patients with TBI.	ENS, EEG.	The stimulus-induced recall of lists was 19% more effective than the non stimulated ones. This discovery provides evidence for closed-loop brain stimulation as a potential therapy for memory impairment caused by TBI.
Longo et al. 2020 [53]	To evaluate the feasibility and safety of LLLT in the acute phase after moderate TBI and neural response to LLLT using MRI and cognitive measures	27 November 2015–11 July 2019.	68 men and women with TBI.	LLLT, RPQ.	LLLT was successfully administered to all patients in this randomized clinical trial without any adverse effects observed. During the late subacute phase, light therapy caused significant changes in several diffusion tensor parameters.
De Freitas et al. 2020 [54]	To see if episodic memory is improved more than just simulated tDCS but enhanced by active tDCS and computer-based cognitive training.	A 20 min. tDCS for 10 days.	36 participants with TBI.	BDI-II, WAIS, RAVLT, AEQ.	The results proved that delta activity decreased and alpha frequencies increased near active electrodes and found a better performance correlation in neuropsychological tests.
Sultana et al. 2023 [55]	To explore the relationship between changes in connectivity and emotional health following rTMS in TBI patients.	20 sessions in 2 weeks.	32 patients with TBI.	VR-36, fMRI.	The results showed an overall decrease in the strength of excitatory connectivity and an increase in the strength of inhibitory connectivity among extrinsic connections after neuromodulation. The central area of analysis was the dorsal anterior cingulate cortex (dACC), which is thought to be most affected during emotional health disorders.
Neville et al. 2019 [56]	To investigate the potential of high-frequency repetitive rTMS to enhance cognitive abilities in individuals who have suffered from severe TBI.	90 days.	Individuals between 18 and 60 years.	TMT-B, rTMS.	Cognitive function in chronic DAI patients does not improve with high-frequency rTMS for the left DLPFC.
Opie et al. 2018 [57]	In this study, TMS and EEG were used further to investigate the impact of mTBI on these processes.	Not Specificated.	32 participants.	GCS, LICI, TMS.	TEP measurements showed that GABA-a and GABA-b activation was not affected by injury; TEP measurements also showed that the response to cTBS was increased in patients, suggesting that cortical plasticity is enhanced due to injury.
Hou et al. 2022 [58]	It investigated the efficacy of TLNS and associated brain connectivity using the RSFC approach in mmTBI patients.	2 weeks.	9 participants with mmTBI.	SOT, DGI.	TLNS in combination with physiotherapy can induce brain plasticity in TBI patients with balance and movement disorders.
Tyler et al. 2019 [59]	The effectiveness of noninvasive TLNS and PT in treating chronic balance/foot gait disorders caused by mmTBI is evaluated through comparison.	26 weeks.	44 Participants	TLNS, PT, SOT.	Balance scores were significantly improved in both the HFP + PT and LFP + PT groups, and the results were maintained for 12 weeks after TLNS treatment discontinuation.

Legend: traumatic brain injury (TBI), external neural stimulator (ENS), electroencephalography (EEG), intermittent theta burst stimulation (iTBS), low-level light therapy (LLLT), magnetic resonance imaging (MRI), Rivermead Post-Concussion Questionnaire (RPQ), transcranial direct current stimulation (tDCS), left dorsolateral prefrontal cortex (lDLPFC), bilateral temporal cortex (BTC), quantitative electroencephalogram (qEEG), Beck Depression Inventory-II (BDI-II), Wechsler Adult Intelligence Scale (WAIS), Rey Auditory Verbal Learning Test (RAVLT), Adverse Events Questionnaire (AEQ), transcranial magnetic stimulation (rTMS), dorsal anterior cingulate cortex (dACC), Veterans RAND 36 Item Health Survey (VR-36), functional magnetic resonance imaging (fMRI), statistical parametric mapping software (SPM 12), Parametric Empirical Bayes (PEB), Trail Making Test-B (TMT-B), left dorsolateral prefrontal cortex (DLPFC), diffuse axonal injury (DAI), mild traumatic brain injury (mTBI), Glasgow Coma Scale (GCS), long-interval intracortical inhibition (LICI), Y-Aminobutyric Acid (GABA), MS-Evoked EEG Potential (TEP), continuous theta burst stimulation (cTBS), electromyography (EMG), translingual neural stimulation (TLNS), mild-to-moderate TBI (mmTBI), resting-state functional connectivity (RSFC), Sensory Organization Test (SOT), Dynamic Gait Index (DGI), physical therapy (PT), high-frequency pulse (HFP), low-frequency pulse (LFP).

## Data Availability

The data that support the findings of this study are not openly available due to reasons of sensitivity and are available from the corresponding author upon reasonable request.
